# Intraregional hospital outbreak of OXA-244-producing *Escherichia coli* ST38 in Norway, 2020

**DOI:** 10.2807/1560-7917.ES.2023.28.27.2200773

**Published:** 2023-07-06

**Authors:** Paul Christoffer Lindemann, Torunn Pedersen, Dorthea Hagen Oma, Jessin Janice, Fredrik Grøvan, Ghantous Milad Chedid, Liv Jorunn Hafne, Ellen H. Josefsen, Oliver Kacelnik, Arnfinn Sundsfjord, Ørjan Samuelsen

**Affiliations:** 1 Department of Microbiology, Haukeland University Hospital, Bergen, Norway; 2 Norwegian National Advisory Unit on Detection of Antimicrobial Resistance, Department of Microbiology and Infection Control, University Hospital of North Norway, Tromsø, Norway; 3 Division of Patient Safety, Haukeland University Hospital, Bergen, Norway; 4 Haraldsplass Deaconess Hospital, Bergen, Norway; 5 Department of Microbiology, Haugesund Hospital, Haugesund, Norway; 6 Department of Antibiotic Resistance and Infection Prevention, Norwegian Institute of Public Health, Oslo, Norway; 7 Department of Medical Biology, UiT The Arctic University of Norway, Tromsø, Norway; 8 Department of Pharmacy, UiT The Arctic University of Norway, Tromsø, Norway

**Keywords:** Carbapenemase, healthcare-associated transmission, high-risk clone, diagnostic challenge

## Abstract

Infections with OXA-244-carbapenemase-producing *Escherichia coli* with sequence type (ST)38 have recently increased in Europe. Due to its low-level activity against carbapenems, OXA-244 can be difficult to detect. Previous assessments have not revealed a clear source and route of transmission for OXA-244-producing *E. coli*, but there are indications of non-healthcare related sources and community spread. Here we report a hospital-associated outbreak of OXA-244-producing *E. coli* ST38 involving three hospitals in Western Norway in 2020. The outbreak occurred over a 5-month period and included 12 cases identified through clinical (n = 6) and screening (n = 6) samples. The transmission chain was unclear; cases were identified in several wards and there was no clear overlap of patient stay. However, all patients had been admitted to the same tertiary hospital in the region, where screening revealed an outbreak in one ward (one clinical case and five screening cases). Outbreak control measures were instigated including contact tracing, isolation, and screening; no further cases were identified in 2021. This outbreak adds another dimension to the spread of OXA-244-producing *E. coli* ST38, illustrating this clone’s ability to establish itself in the healthcare setting. Awareness of challenges concerning OXA-244-producing *E. coli* diagnostic is important to prevent further spread.

**Key public health message**

**What did you want to address in this study?**

Bacteria capable of resisting antibiotic drugs pose a problem for healthcare. Prevalence of antibiotic resistance is low in Norway. Among several ways that bacteria can protect themselves against antibiotics and develop resistance, one is production of a protein called carbapenemase. Here, we wanted to understand and control an outbreak affecting several hospitals in Western Norway, which was due to a multidrug-resistant bacterial strain that produced carbapenemase.

**What have we learnt from this study?**

The outbreak strain was an *Escherichia coli* bacterium; it had genetic sequence type ST38 and it produced a carbapenemase named OXA-244 which is hard-to-detect by routine diagnostic. Contact tracing of patients infected with (or carrying) the outbreak strain, screening, and infection-control measures’ reinforcement contributed to limiting the outbreak. The outbreak strain’s features challenged microbiological diagnostics, especially semi-automated methods.

**What are the implications of your findings for public health?**

Recently, OXA-244-producing *E. coli* ST38 infections have increased in Europe and seemed to involve community transmission. This Norwegian intraregional healthcare-associated outbreak adds another aspect to the spread of this high-risk clone. Finding neither the outbreak source nor a clear transmission chain warrants investigating the role of non-healthcare related origins. Continuous national surveillance to detect cross-hospital-outbreak signals is important.

## Background

Oxacillinase-48 (OXA-48)-type β-lactamases have become the dominant carbapenemase among carbapenemase-producing *Enterobacterales* (CPE) in certain areas of the world [1]. Their rapid dissemination is linked to an epidemic IncL plasmid and/or association with specific high-risk clones [1]. Several variants of OXA-48 have been described including OXA-244 which is a one amino-acid variant of OXA-48 (OXA-48 Arg214Gly) with reduced activity against carbapenems and temocillin [2], posing diagnostic challenges [3,4]. Since around 2013 and onwards, an increase in OXA-244-producing *Escherichia coli* has been observed in several European countries [5-10]. Genomic studies show that the dissemination of OXA-244-producing *E. coli* is polyclonal, but dominated by *E. coli* sequence type (ST) 38 [5,8-10]. Previous studies indicate that the dissemination of OXA-244-producing *E. coli* ST38 is mainly associated with non-healthcare transmission or import [5,8-10]. Here we report an outbreak of OXA-244-producing *E. coli* ST38 involving three hospitals in the Western region of Norway, a country with a very low prevalence of CPE [11,12].

### Outbreak detection

In July 2020, two putative carbapenemase-producing clinical isolates of *E. coli* were submitted from two different hospitals (Hospital 1 and Hospital 2) in Western Norway to the Norwegian National Advisory Unit on Detection of Antimicrobial Resistance, which is the national reference laboratory for carbapenemase-producing Gram-negative bacteria. In both cases, no association with import was suspected. The initial analysis confirmed that the isolates were *bla*_OXA-48-like_ positive by PCR and had a similar resistance profile. Both hospitals were notified of the possibility of regional transmission. Epidemiological investigation revealed that both patients had been admitted to the same ward at Hospital 2, at different time points, which signalled a possible outbreak. 

Here, we report the epidemiology, infection control measures, and microbial characteristics of the ensuing outbreak and compare the outbreak strain with other OXA-244-producing *E. coli* ST38 from other regions in Norway.

## Methods

### Case definition

Cases were defined as patients infected or colonised with OXA-244-producing *E. coli* ST38 hospitalised at the affected hospitals in Western Norway between July and November 2020. 

### Identification of outbreak cases

Cases were identified either through routine diagnostic analysis of clinical samples or rectal screening for CPE including routine contact tracing to identify secondary cases. Contacts were defined as patients co-hospitalised in the same ward as cases*.* Weekly surveillance and rectal swabbing of all patients in affected wards were implemented. 

To investigate potential interregional spread, we also included genomic data for all other identified OXA-244-producing *E. coli* ST38 from other regions in Norway in 2020. Some of these data are described in Supplementary Table 1.

### Microbiological investigations

Rectal swabs were inoculated on CHROMagar mSuperCARBA agar plates (CHROMagar, Paris, France) after confirming that the medium supported the growth of the outbreak strain. Species were identified using the MALDI Biotyper (Bruker Daltonics, Bremen, Germany). Antimicrobial susceptibility testing was performed according to the European Committee on Antimicrobial Susceptibility Testing (EUCAST) disc diffusion method, broth microdilution (Sensititre, Trek Diagnostic Systems/Thermo Fisher Scientific, East Grindstead, United Kingdom) and VITEK2 using the AST-N384 card (bioMérieux, Marcy-l'Étoile, France) and interpreted according to EUCAST clinical breakpoints v.13 [13]. Phenotypic carbapenemase-production were investigated using the KPC, MBL, and OXA-48 confirm kit (Rosco, Taastrup, Denmark) and β-CARBA test (Bio-Rad, Marnes-la-Coquette, France).

### Molecular analysis and whole genome sequencing

Detection of carbapenemase genes was done using multiplex PCR [14]. DNA from *bla*_OXA-48_-like positive isolates was extracted using the automated EZ1 platform (Qiagen, Hilden, Germany). DNA libraries generated using the Nextera kit were sequenced by Illumina technology on the Illumina NextSeq550/MiSeq platforms (Illumina, San Diego, United States (US)). For Oxford Nanopore Technologies (ONT; Oxford, England) sequencing, DNA was further purified by AMPure XP beads (A63882; Beckman Coulter, Krefeld, Germany). ONT libraries were generated by Rapid Barcoding kit (ONT; SQK-RBK001) and sequencing on MinION Mk1C using R9.4 flow cells (FLO-MIN106). Antimicrobial resistance genes were detected using AMRFinderPlus v. 3.10.1. Illumina sequences were assembled using SPAdes v.3.12.0. Hybrid assemblies were constructed from ONT and Illumina fastq files using Unicycler v.0.4.8 in normal mode. Phylogeny was performed using the Ridom SeqSphere+ software (Ridom, Münster, Germany) for core genome multilocus sequence typing (cgMLST) with the integrated cgMLST scheme and *E. coli* K12 as reference genome (GenBank accession number: NC_000913.3).

## Results

A total of 12 cases of *bla*_OXA-48-like_ positive *E. coli* were identified from July 2020 to November 2020 (Table 1); the median age was 68.5 years (range: 47–87 years) with an equal number of males and females. Following the first two cases in July 2020 at Hospitals 1 and 2, another two clinical isolates (cases 3 and 4) were detected in August 2020 from separate wards at Hospital 2. Contact tracing around case 4 uncovered a local outbreak at Surgical ward #2 as weekly routine screening of all admitted patients detected five more carriers (cases 5–7, 9, and 12) over a period of 12 weeks (September to November 2020). Three of those had shared a room with one of the previously identified cases (case 4). Moreover, in September 2020 a clinical isolate from urine was detected at Hospital 3 (case 8). This patient had been admitted to Hospital 2 for a few days in June/July 2020, but at a different ward than the other cases. Finally, two randomly detected isolates were found during October 2020. One isolate (case 10) was detected by routine extended spectrum β-lactamase (ESBL)/carbapenemase rectal carriage screening on admission to the intensive care unit and one (case 11) was detected in a urine sample taken at the emergency department, both at Hospital 2. Both case 10 and case 11 had recently been admitted to gastroenterology and the pulmonary medicine ward at Hospital 2, respectively. Contact tracing following case 10 and 11 revealed no secondary cases. Association with import was not suspected for any cases.

**Table 1:** Overview of outbreak cases identified with OXA-244-producing *Escherichia coli* ST38 in the Western region of Norway, 2020 (n = 12)

Case number	Isolate ID.	Sample date (month/year)	Sample material	Hospital number^a^	Hospital ward^a^	Age group in years
1	KresCPE0297	07/2020	Urine	1	Internal medicine ward	41–50
2	KresCPE0296	07/2020	Drainage fluid	2	Surgical ward #1	61–70
3	KresCPE0301	08/2020	Urine	2	Pulmonary medicine – outpatient	51–60
4	KresCPE0304	08/2020	Drainage fluid	2	Surgical ward #2	61–70
5	KresCPE0306	09/2020	Rectal swab	2	Surgical ward #2	51–60
6	KresCPE0307	09/2020	Rectal swab	2	Surgical ward #2	81–90
7	KresCPE0308	09/2020	Rectal swab	2	Surgical ward #2	61–70
8	KresCPE0310	09/2020	Urine	3	Medicine ward	81–90
9	KresCPE0313	10/2020	Rectal swab	2	Surgical ward #2	71–80
10	KresCPE0314	10/2020	Rectal swab	2	Intensive care unit	51–60
11	KresCPE0316	10/2020	Urine	2	Emergency department	61–70
12	KresCPE0326	11/2020	Rectal swab	2	Surgical ward #2	71–80

ID: identifier; OXA: oxacillinase.

^a^ At sample date.

### Genetic characterisation and comparison of OXA-244-producing *Escherichia coli* ST38 isolates

**Figure 1:**
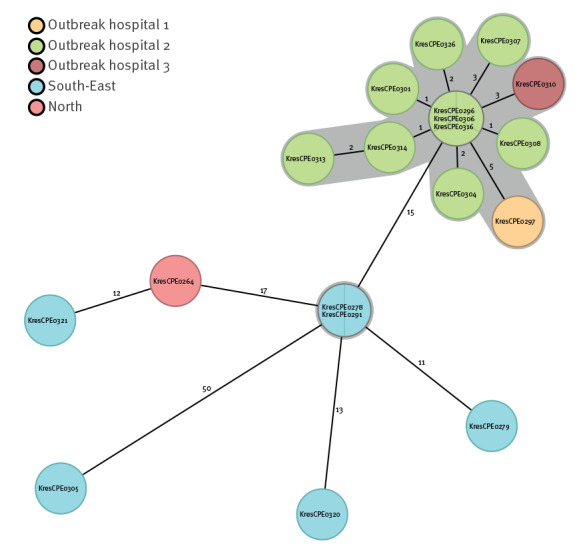
Minimum spanning tree based on the core genome allele profile of OXA-244-producing *Escherichia coli* ST38 outbreak isolates (n = 12) from Western Norway and isolates from other regions in Norway in 2020 (n = 7)

OXA: oxacillinase; MLST: multilocus sequence typing; ST: sequence type.

Whole genome sequencing of the outbreak isolates showed that all isolates belonged to ST38, cgMLST cluster type 8582 and harboured *bla*_OXA-244_, as described in Supplementary Table 1. Phylogenetic analysis using cgMLST confirmed that they were closely related, differing by 0–8 alleles (Figure 1). Comparison with other OXA-244-producing *E. coli* ST38 isolates from Norway in 2020, shown in Supplementary Table 1, confirmed the intraregional nature of the outbreak (Figure 1). No other isolates belonged to the same cluster type as the outbreak isolates and 15–62 cgMLST allelic differences were found between the outbreak and non-outbreak isolates. Among isolates not belonging to the outbreak, two OXA-244-producing *E. coli* ST38, which both belonged to cluster type 2883 showed no cgMLST allele differences. However, these were from two different laboratories and no epidemiological link between the isolates could be established. The outbreak was notified to the European Centre for Disease Prevention and Control (ECDC) through the EpiPulse surveillance portal resulting in a genomic analysis of 370 OXA-244-producing *E. coli* ST38 isolates mainly from European countries [10]. This analysis showed that the Norwegian outbreak isolates were part of a large cluster with ≤ 20 cgMLST allelic differences containing 225 isolates from 12 European countries and in general a conserved set of resistance genes. Within this cluster the Norwegian outbreak isolates formed a separate subcluster with the closest related, being an isolate from the Netherlands also from 2020.

Each isolate is represented by a circle coloured according to the hospital involved in the outbreak or region. The number of core genome MLST allele differences between isolates is indicated with a number along the connecting lines between circles. Circles containing > 1 isolate indicate no core genome MLST allele differences. Grey shading show clusters of isolates with a core genome MLST allele difference below the cluster threshold (≤ 10 allele differences).

**Figure 2:**
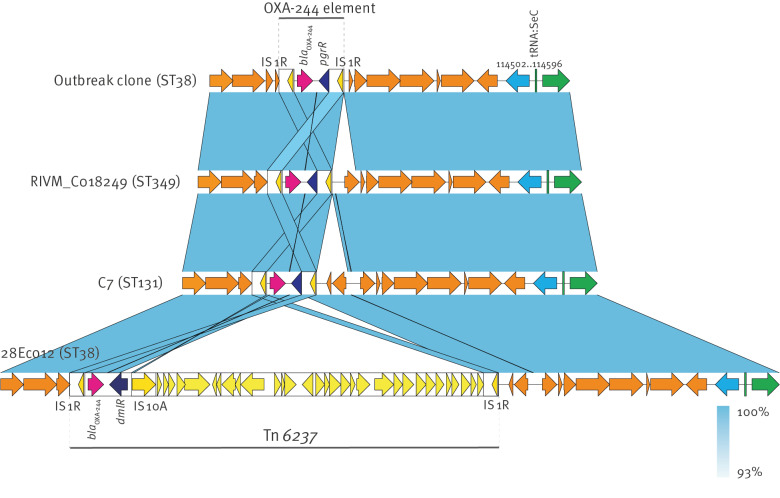
Comparisons of the *bla*_OXA-244_ containing chromosomal region in the outbreak clone with selected isolates of ST349, ST131 and ST38 representing three variants

OXA: oxacillinase; RNA: ribonucleic acid; ST: sequence type.

The chromosomal position of *bla*_OXA-244_ in the ST38 outbreak clone is consistent with the findings of previous investigations, which locate *bla*_OXA-48_-like genes on IS*1R*-flanked composite elements of different sizes [15-17]. As illustrated in Figure 2, *bla*_OXA-244_ is together with *pgrR* encoding a transcriptional regulator [18], bracketed by IS*1R*. We detected similar *bla*_OXA-244_ elements in completed *E. coli* genomes of ST38 and ST349 from the Netherlands (BioProjects PRJNA774636 and PRJNA691727), ST131 from Italy (NZ_CP059279.1 and NZ_CP059281.1), and ST167 from the Czech Republic (NZ_CP050382.1). However, sequence comparisons including surrounding chromosomal regions divided them into three different variants (Figure 2). Alignment with Tn*6237*, located to a genomic island inserted into tRNA:SeC of 28Eco12 [15], indicates that these variants are remnants of Tn*6237* and the genomic variation is due to partial loss of the flanking genomic island. Notably, *pgrR* is a truncated version of the LysR type regulator *dmlR* [19].

The GenBank accession for ST349 is NZ_CP068802.1, for ST131 NZ_CP059279.1 and for ST38 NZ CP038505.1. The chromosomal position of the OXA-244-encoding element (3,308 bp) upstream of tRNA:SeC is indicated. Arrows represent annotated coding sequences with *bla*_OXA-244_ (fuchsia), the transcription regulators *pgrR* and *dmlR* (dark blue), prophage integrase *intS* (turquoise), with Tn*6237* (yellow), genomic island (orange) and *E. coli* (green) genes highlighted. Mobile elements are boxed (white). Blue shading between pairs of sequences indicates identity (93 to 100%) as indicated by the colour code.

A conserved set of additional resistance genes was found in the outbreak isolates. This included *bla*_CTX-M-27_, *aadA5, aph(3”)-Ib, aph(6)-Id, dfrA17*, *mph(A)*, *tetA*, *sul1,* and *sul2* and was consistent with the antimicrobial susceptibility profile (Table 2). Using long-read sequencing, we obtained a complete, highly conserved putative conjugative IncFII/IncFIB plasmid (ca 148 kbp) for eight of the 12 outbreak cases’ strain as illustrated in Supplementary Figure 1. Further, mapping of the genomic sequences to the reference plasmid of the remaining four outbreak strains indicated the presence of the plasmid in these.

**Table 2:** Antimicrobial susceptibility of OXA-244-producing *Escherichia coli* ST38 outbreak isolates in the Western region of Norway, 2020 (n = 12)

Isolate ID	Minimum inhibitory concentration (MIC, mg/L)^a,b^																				
	TEM	PIT	CTA	CTZ	CTV	CTT	CEP	CEF^a^	AZT	ERT	IMI	MEM	MEV	AMI	GEN	TOB	CIP	COL	TIG	TRS	NIT
KresCPE0297	32	64	> 16	4	0.12	2	4	22	8	1	0.25	0.25	0.12	< 1	< 0.25	< 0.5	< 0.015	1	0.25	> 16	32
KresCPE0296	64	> 64	> 16	8	0.12	4	4	24	8	2	0.25	0.25	0.25	< 1	< 0.25	< 0.5	< 0.015	1	< 0.12	> 16	32
KresCPE0301	128	> 64	> 16	8	0.12	8	8	23	16	2	0.25	0.5	0.25	< 1	< 0.25	< 0.5	< 0.015	1	< 0.12	> 16	32
KresCPE0304	128	> 64	> 16	8	0.12	8	8	23	8	4	0.5	1	0.5	< 1	< 0.25	< 0.5	< 0.015	1	< 0.12	> 16	< 16
KresCPE0306	32	64	> 16	4	0.12	2	8	24	8	1	0.25	0.25	0.12	< 1	< 0.25	< 0.5	< 0.015	1	< 0.12	> 16	32
KresCPE0307	128	> 64	> 16	4	0.12	8	8	25	16	4	0.5	0.5	0.5	< 1	0.5	< 0.5	< 0.015	1	< 0.12	> 16	< 16
KresCPE0308	128	> 64	> 16	4	0.12	8	8	22	8	4	0.5	0.5	0.5	< 1	< 0.25	< 0.5	< 0.015	1	< 0.12	> 16	32
KresCPE0310	32	64	> 16	4	< 0.03	1	4	25	8	1	0.12	0.25	0.25	< 1	< 0.25	< 0.5	< 0.015	0.5	< 0.12	> 16	< 16
KresCPE0313	32	64	> 16	2	< 0.03	2	2	27	4	2	0.12	0.5	0.12	< 1	< 0.25	< 0.5	< 0.015	1	0.25	> 16	< 16
KresCPE0314	128	> 64	> 16	4	0.12	4	8	22	8	4	1	1	0.5	< 1	< 0.25	< 0.5	< 0.015	1	< 0.12	> 16	32
KresCPE0316	32	64	> 16	4	0.12	2	8	23	16	1	0.25	0.25	0.12	< 1	< 0.25	< 0.5	< 0.015	0.5	0.25	> 16	32
KresCPE0326	32	> 64	> 16	4	0.12	2	4	22	8	1	0.25	0.25	0.5	< 1	0.5	< 0.5	< 0.015	1	< 0.12	> 16	32

ID: identifier; MIC: minimum inhibitory concentration; OXA: oxacillinase; ST: sequence type.

^a^ MIC data generated by broth microdilution, except for CEF where millimetre zone diameters from disc diffusion are presented.

^b^ AMI: amikacin; AZT: aztreonam; CTA: cefotaxime; CEF: cefiderocol; CEP: cefepime; CIP: ciprofloxacin; COL: colistin; CTV: ceftazidime–avibactam; CTT: ceftolozane–tazobactam; CTZ: ceftazidime; ERT: ertapenem; GEN: gentamicin; IMI: imipenem; MEM: meropenem; MEV: meropenem–vaborbactam; NIT: nitrofurantoin; PIT: piperacillin–tazobactam; TEM: temocillin; TIG: tigecycline; TOB: tobramycin; TRS: trimethoprim–sulfamethoxazole.

### Phenotypic detection of OXA-244-producing *Escherichia coli*

OXA-244 poses a phenotypic diagnostic challenge due to lower activity against carbapenems and temocillin which is used as an indicator for the presence of OXA-48-like carbapenemases [2-4]. All isolates showed minimum inhibitory concentrations (MIC)s or zone diameters (Table 2) within the EUCAST meropenem screening criteria for carbapenemase-production [13]; this is also described in Supplementary Table 2. All isolates were also resistant to piperacillin–tazobactam fulfilling the Nordic Committee on Antimicrobial Susceptibility Testing (NordicAST) criteria for carbapenemase screening of isolates with meropenem zone diameters of 25–27 mm [20]. However, none of the isolates showed high-level temocillin resistance (MIC > 128 mg/L). Using the Rosco KPC, MBL and OXA-48 confirm kit, synergy was not observed between meropenem and β-lactamase inhibitors for any isolates. Only four isolates did have a temocillin zone diameter of < 12 mm indicative of OXA-48 according to NordicAST guidelines [20]. Interestingly, six isolates showed a meropenem MIC of ≤ 0.25 mg/L on VITEK2 and were interpreted by the expert system as ESBL-positive, as detailed in Supplementary Table 2. All isolates were positive for carbapenemase-production by the β-CARBA test.

### Outbreak control measures

Contact tracing and weekly rectal screening of all patients in the affected wards were initiated in all three hospitals. The control measures uncovered the local outbreak in Surgical ward #2 and the standard screening procedures revealed an additional case in the intensive care unit. Surgical ward #2 also focused on contact isolation of inpatients, antibiotic stewardship, reinforcement of standard precautions including hand hygiene practice (healthcare workers and inpatients), and environmental cleaning and disinfection. The outbreak was notified to the National Institute of Public Health and information on it published in various channels to alert diagnostic laboratories in Norway. The national reference laboratory further notified laboratories in Norway about challenges in the detection of the outbreak clone and shared the outbreak clone with other laboratories for quality control purposes. The continuous surveillance of CPE in Norway by whole genome sequencing also allowed comparative phylogenetic analysis of the outbreak clone with other OXA-244-producing *E. coli* ST38 identified at other laboratories revealing no interregional spread. The outbreak was also notified to ECDC resulting in an update of a previous assessment of the spread of OXA-244-producing *E. coli* in Europe [10].

## Discussion

Norway is a low-prevalence country in terms of CPE with 30–75 reported cases per year in the last 5 years [21]. Most cases are associated with travel outside of Norway [22]. Annual whole-genome sequence-based surveillance data indicate limited within-country spread of CPE [21]. Except for a small regional outbreak of *K. pneumoniae* carbapenemase (KPC)-producing *K. pneumoniae* in 2007–2010, no major outbreaks have been identified [23]. Here, we describe the largest outbreak of CPE in Norway reported so far, which involved 12 cases and three hospitals.

The outbreak clone belongs to ST38, a common extra-intestinal pathogenic *E. coli* clone associated with antimicrobial resistance [24]. In Europe, the observed increase in regional and national dissemination of OXA-244-producing *E. coli* ST38 has mainly been associated with transmission in the community [5,8]. However, our findings demonstrate the potential of healthcare-associated transmission of OXA-244-producing *E. coli* ST38 once introduced into the hospital setting.

The initial source of the outbreak could not be identified. Neither of the first two detected cases had a known travel history which is unusual in a Norwegian context. Moreover, a challenging aspect of the outbreak was the identification of cases in multiple departments and at three hospitals. Since no clear epidemiological link could be established for some of the cases an unknown reservoir or undetected cases could be possible despite the contact tracing and screening instigated. One limitation of the study is the lack of environmental screening. In addition, healthcare workers were not screened according to national guidelines. However, the implemented infection control measures were successful since no further cases were identified at the respective hospitals in 2021.

A high level of diagnostic awareness is important for OXA-244 producers. Evaluation of chromogenic screening agars shows poor sensitivity for some CPE-specific media for detection of OXA-244 producers [3,4,7]. Due to the presence of an ESBL (CTX-M-27) in the outbreak clone, we could have used a chromogenic ESBL media for the rectal sample screening. However, possible loss of the CTX-M plasmid could have resulted in undetected screening cases of non-ESBL-producing OXA-244 isolates. Regardless, laboratories should be aware of the challenges in phenotypic detection of OXA-244 producers to prevent their spread and establishment in the healthcare setting. The VITEK2 misinterpretation of six strains (50%) as non-carbapenemase producers raises awareness of the potential occurrence of false negatives when using of semi-automated methods for the detection of CPE with non-wildtype low meropenem-MICs. One limitation with the analysis is the use of a broth microdilution method that deviates slightly from the International Organization for Standardization (ISO) standard [25].

### Conclusions

This healthcare-associated outbreak of OXA-244-producing *E. coli* ST38 adds another dimension to the previously described community spread of this high-risk clone. The initial outbreak identification underlines the importance of national surveillance of CPE enabling outbreak detection despite isolates being identified in different laboratories. The non-wildtype low meropenem MICs and moderately elevated temocillin MICs associated with OXA-244 *E. coli* challenge diagnostic CPE-screening criteria and the use of semi-automated antimicrobial susceptibility methods in CPE detection.

## Supplementary Data

481000 Supplementary Material
