# Supplementary material for: Intraregional hospital outbreak of OXA-244-producing Escherichia coli ST38 in Norway, 2020
Source: Euro Surveill. 2023 Jul 6;28(27):2200773. doi: 10.2807/1560-7917.ES.2023.28.27.2200773 (PMC10370041; doi:10.2807/1560-7917.ES.2023.28.27.2200773)
Supplement: Supplementary Material [file 22-00773_SupplementaryMaterial.pdf]

Supplementary Material:
This supplementary material is hosted by Eurosurveillance as supporting information alongside the article Intraregional hospital outbreak of OXA-244-producing *Escherichia coli* ST38 in Norway, 2020, on behalf of the authors, who remain responsible for the accuracy and appropriateness of the content. The same standards for ethics, copyright, attributions and permissions as for the article apply. Supplements are not edited by Eurosurveillance and the journal is not responsible for the maintenance of any links or email addresses provided therein.

Supplementary Table 1. OXA-244-producing *E. coli* ST38 outbreak isolates from the Western region (n=12) and isolates identified in Norway outside the Western region in 2020 (n=7).

**Isolate ID.**	**Region**	**cgMLST cluster type**	**Antimicrobial resistance genes**
KresCPE0297	West	8582	$bla_{OXA-244}$, $bla_{CTX-M-27}$, *aph(3'')-Ib, aph(6)-Id, aadA5, dfrA17, sul1, sul2*
KresCPE0296	West	8582	$bla_{OXA-244}$, $bla_{CTX-M-27}$, *aph(3'')-Ib, aph(6)-Id, aadA5, dfrA17, sul1, sul2*
KresCPE0301	West	8582	$bla_{OXA-244}$, $bla_{CTX-M-27}$, *aph(3'')-Ib, aph(6)-Id, aadA5, dfrA17, sul1, sul2*
KresCPE0304	West	8582	$bla_{OXA-244}$, $bla_{CTX-M-27}$, *aph(3'')-Ib, aph(6)-Id, aadA5, dfrA17, sul1, sul2*
KresCPE0306	West	8582	$bla_{OXA-244}$, $bla_{CTX-M-27}$, *aph(3'')-Ib, aph(6)-Id, aadA5, dfrA17, sul1, sul2*
KresCPE0307	West	8582	$bla_{OXA-244}$, $bla_{CTX-M-27}$, *aph(3'')-Ib, aph(6)-Id, aadA5, dfrA17, sul1, sul2*
KresCPE0308	West	8582	$bla_{OXA-244}$, $bla_{CTX-M-27}$, *aph(3'')-Ib, aph(6)-Id, aadA5, dfrA17, sul1, sul2*
KresCPE0310	West	8582	$bla_{OXA-244}$, $bla_{CTX-M-27}$, *aph(3'')-Ib, aph(6)-Id, aadA5, dfrA17, sul1, sul2*
KresCPE0313	West	8582	$bla_{OXA-244}$, $bla_{CTX-M-27}$, *aph(3'')-Ib, aph(6)-Id, aadA5, dfrA17, sul1, sul2*
KresCPE0314	West	8582	$bla_{OXA-244}$, $bla_{CTX-M-27}$, *aph(3'')-Ib, aph(6)-Id, aadA5, dfrA17, sul1, sul2*
KresCPE0316	West	8582	$bla_{OXA-244}$, $bla_{CTX-M-27}$, *aph(3'')-Ib, aph(6)-Id, aadA5, dfrA17, sul1, sul2*
KresCPE0326	West	8582	$bla_{OXA-244}$, $bla_{CTX-M-27}$, *aph(3'')-Ib, aph(6)-Id, aadA5, dfrA17, sul1, sul2*
KresCPE0264	North	6448	$bla_{OXA-244}$, $bla_{CTX-M-27}$, *aph(3'')-Ib, aph(6)-Id, aadA5, dfrA17, sul1, sul2*
KresCPE0278	South-East	2883	$bla_{OXA-244}$, $bla_{CTX-M-27}$, *aph(3'')-Ib, aph(6)-Id, aadA5, dfrA17, sul1, sul2*
KresCPE0279	South-East	2883	$bla_{OXA-244}$, $bla_{CTX-M-27}$, *aph(3'')-Ib, aph(6)-Id, sul2*
KresCPE0291	South-East	2883	$bla_{OXA-244}$, $bla_{CTX-M-27}$, *aph(3'')-Ib, aph(6)-Id, aadA5, dfrA17, sul1, sul2*
KresCPE0305	South-East	11109	$bla_{OXA-244}$, bla_{TEM-1}, *aph(3'')-Ib, aph(6)-Id, sul2*
KresCPE0320	South-East	11110	$bla_{OXA-244}$, $bla_{CTX-M-27}$, *aph(3'')-Ib, aph(6)-Id, aadA5, dfrA17, sul1, sul2*
KresCPE0321	South-East	6448	$bla_{OXA-244}$, $bla_{CTX-M-27}$, *aph(3'')-Ib, aph(6)-Id, dfrA17, sul1, sul2*

Supplementary Table 2. Comparison of phenotypic results of OXA-244-producing *E. coli* ST38 outbreak isolates, Western Norway, 2020 (n=12).

Isolate ID	EUCAST meropenem disc diffusion zone diameter (mm)	Zone diameter (mm) ROSCO KPC, MBL and OXA-48 confirm kit[1]					β-CARBA test	Broth microdilution meropenem MIC (mg/L)	VITEK2 meropenem MIC (mg/L)	VITEK2 interpretation[2]
		TEMOC	MRP10	MRPBO	MRPCX	MRPDP				
KresCPE0297	24	14	26	25	26	26	Positive	0.25	≤0.25	ESBL
KresCPE0296	23	12	26	25	25	26	Positive	0..25	0.5	Carbapenemase (+ or – ESBL), Impermeability (? ESBL or + HL AmpC)
KresCPE0301	23	9	25	25	24	24	Positive	0.5	0.5	Carbapenemase (+ or – ESBL), Impermeability (? ESBL or + HL AmpC)
KresCPE0304	22	9	21	21	22	22	Positive	1	1	Carbapenemase (+ or – ESBL)
KresCPE0306	26	14	27	27	27	28	Positive	0.25	≤0.25	ESBL
KresCPE0307	22	9	23	23	23	21	Positive	0.5	1	Carbapenemase (+ or – ESBL)
KresCPE0308	22	9	22	23	23	22	Positive	0.5	1	Carbapenemase (+ or – ESBL)
KresCPE0310	23	17	26	26	27	27	Positive	0.25	≤0.25	ESBL
KresCPE0313	24	14	27	27	27	27	Positive	0.5	≤0.25	ESBL
KresCPE0314	22	12	23	24	24	23	Positive	1	1	Carbapenemase (+ or – ESBL)
KresCPE0316	25	14	25	26	26	25	Positive	0.25	≤0.25	ESBL
KresCPE0326	25	14	22	22	22	21	Positive	0.25	≤0.25	ESBL

[1]TEMOC: temocillin 30µg; MRP10: meropenem 10µg; MRPBO: meropenem 10µg + phenylboronic acid; MRPCX: meropenem 10µg + cloxacillin; MRPDP: meropenem 10µg + dipicolinic acid. 2 ESBL: extended-spectrum β-lactamase; HL: high-level.

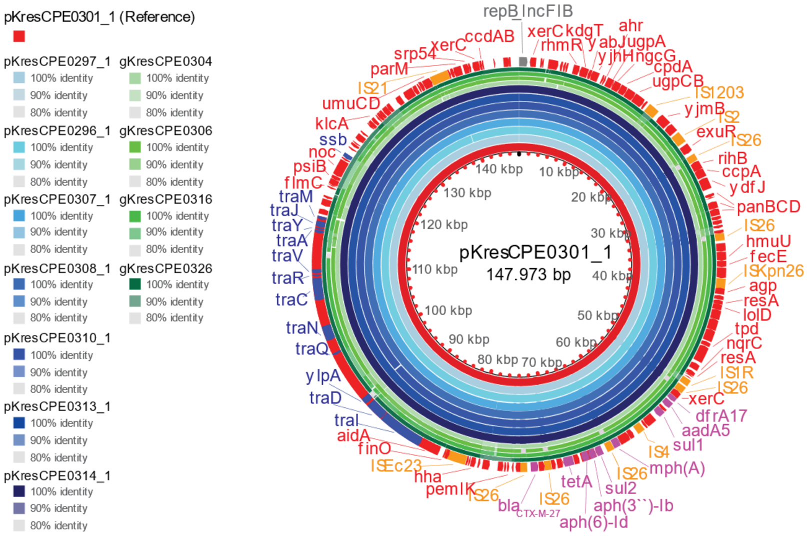

Supplementary Figure 1. BLAST comparisons of the IncFII/IncFIB resistance plasmid from KresCPE0301 (case 3) with corresponding plasmids (p) or total genomic DNA (g) from the *E. coli* ST38-OXA-244 outbreak strains (n=12) from Western Norway, 2020 visualized by the concentric circles as listed. The given colour codes indicate DNA identity (80-100%) with missing regions appearing white. Annotated coding sequences (red arrows) are shown in the outermost circle with resistance (lilac), *tra* genes involved in conjugative transfer (blue), and IS-elements (orange) highlighted. The map was constructed using the BRIG software (http://brig.sourceforge.net/).
